# *Chd8* haploinsufficiency impairs early brain development and protein homeostasis later in life

**DOI:** 10.1186/s13229-020-00369-8

**Published:** 2020-10-05

**Authors:** Jessica A. Jiménez, Travis S. Ptacek, Alex H. Tuttle, Ralf S. Schmid, Sheryl S. Moy, Jeremy M. Simon, Mark J. Zylka

**Affiliations:** 1 Curriculum in Toxicology & Environmental Medicine, The University of North Carolina at Chapel Hill, Chapel Hill, NC 27599 USA; 2 UNC Neuroscience Center, The University of North Carolina at Chapel Hill, Chapel Hill, NC 27599 USA; 3 Carolina Institute for Developmental Disabilities, The University of North Carolina at Chapel Hill, Chapel Hill, NC 27599 USA; 4 Department of Psychiatry, The University of North Carolina at Chapel Hill, Chapel Hill, NC 27599 USA; 5 Department of Genetics, The University of North Carolina at Chapel Hill, Campus Box #7264, Chapel Hill, NC 27599 USA; 6 Department of Cell Biology and Physiology, The University of North Carolina at Chapel Hill, Chapel Hill, NC 27599 USA

**Keywords:** CHD8, Macrocephaly, Brain overgrowth, Autism spectrum disorder, Unfolded protein response, Endoplasmic reticulum stress

## Abstract

**Background:**

Chromodomain helicase DNA-binding protein 8 (*Chd8)* is a high-confidence risk gene for autism spectrum disorder (ASD). However, how *Chd8* haploinsufficiency impairs gene expression in the brain and impacts behavior at different stages of life is unknown.

**Methods:**

We generated a mutant mouse line with an ASD-linked loss-of-function mutation in *Chd8* (V986*; stop codon mutation). We examined the behavior of *Chd8* mutant mice along with transcriptional changes in the cerebral cortex as a function of age, with a focus on one embryonic (E14.5) and three postnatal ages (1, 6, and 12 months).

**Results:**

*Chd8*^V986*/+^ mutant mice displayed macrocephaly, reduced rearing responses and reduced center time in the open field, and enhanced social novelty preference. Behavioral phenotypes were more evident in *Chd8*^V986*/+^ mutant mice at 1 year of age. Pup survival was reduced in wild-type x *Chd8*^V986*/+^ crosses when the mutant parent was female. Transcriptomic analyses indicated that pathways associated with synaptic and neuronal projections and sodium channel activity were reduced in the cortex of embryonic *Chd8*^V986*/+^ mice and then equalized relative to wild-type mice in the postnatal period. At 12 months of age, expression of genes associated with endoplasmic reticulum (ER) stress, chaperone-mediated protein folding, and the unfolded protein response (UPR) were reduced in *Chd8*^V986*/+^ mice, whereas genes associated with the c-MET signaling pathway were increased in expression.

**Limitations:**

It is unclear whether the transcriptional changes observed with age in *Chd8*^V986*/+^ mice reflect a direct effect of CHD8-regulated gene expression, or if CHD8 indirectly affects the expression of UPR/ER stress genes in adult mice as a consequence of neurodevelopmental abnormalities.

**Conclusions:**

Collectively, these data suggest that UPR/ER stress pathways are reduced in the cerebral cortex of aged *Chd8*^V986*/+^ mice. Our study uncovers neurodevelopmental and age-related phenotypes in *Chd8*^V986*/+^ mice and highlights the importance of controlling for age when studying *Chd8* haploinsufficient mice.

## Background

Autism spectrum disorder (ASD) is characterized by social deficits and repetitive/restricted behaviors and affects approximately 0.6% of the global population [1]. Recent whole exome and whole genome sequencing studies identified various ASD risk alleles, but the underlying biology and disease progression associated with these mutations remain poorly understood [2–6].

*Chromodomain helicase DNA-binding protein 8* (*CHD8)* encodes an ATP-dependent chromatin remodeler. An association between *CHD8* and ASD was first identified in two patients with a microdeletion encompassing *CHD8* and *SUPT16H* [7]. Subsequent studies found that de novo heterozygous loss-of-function mutations in *CHD8* are relatively common in ASD probands [4, 8–12]. Individuals with disruptive *CHD8* mutations are disproportionately male (3.5:1) and present with shared symptoms, including macrocephaly, neonatal hypotonia, distinct facial features, and gastrointestinal problems [9, 13, 14].

Mice carrying heterozygous mutations in *Chd8* display various ASD-related phenotypes, including macrocephaly, repetitive behavior, and cognitive impairments [15–20]. However, phenotypes in these various *Chd8* haploinsufficiency mouse lines are only partially concordant and are of small effect size. A recent study found that phenotypes were only detected in male *Chd8* mutant mice [20], suggesting penetrance may be influenced by sex, as is the case in humans [13]. *CHD8* disruption affects genes associated with neurodevelopment and synaptic function [21–24]*.* Transcriptome profiling in *Chd8* mutant mice revealed alterations, albeit subtle, in cellular processes including cell-cycle regulation, transcription, development, histone/chromatin modification, Wnt signaling, neurogenesis, and synaptogenesis [15, 18, 19]. Whether any of these differentially expressed genes are shared between the different *Chd8* mutant mouse lines has never been evaluated.

Thus far, studies with *Chd8* mutant mice focused on embryonic, early postnatal, and young adult time points. However, two landmark transcriptomic studies by Voineagu et al. and Gupta et al. made use of postmortem ASD brain samples from individuals with average ages of 24.0 (range 5–51) and 23.5 (range 2–82), respectively [25, 26]. Both of these studies reported an increase in immune-related genes and glial genes in individuals with ASD [25, 26]. Curiously, however, this neuroimmune signature has never been detected in ASD mouse models. One possible reason for this discrepancy could relate to the relatively young age at which mouse ASD models have been studied. Little is currently known about symptoms in older individuals with ASD [27, 28], highlighting a major gap in the field. To evaluate age as a variable in a high-confidence ASD mouse model and to facilitate the identification of shared behavioral and transcriptomic phenotypes that result from *Chd8* haploinsufficiency, we generated a new mouse line with an ASD-linked loss-of-function mutation in *Chd8* (V986*). We examined behavioral phenotypes at two ages and examined gene expression in the cerebral cortex at an embryonic time point and at three postnatal time points. We found that behavioral phenotypes were more evident with age and that distinct molecular pathways are affected in the brain of *Chd8*^V986*/+^ mice at embryonic versus older adult (1 year) ages. Collectively, our study highlights the importance of expanding the age range over which ASD model mice are evaluated.

## Methods

### Mice

Mice were maintained on a C57BL/6J background and all experiments used littermate controls. Mice were raised in a facility with a 12:12-light:dark cycle with ad libitum access to food (Teklad 2020X, Envigo, Huntingdon, UK) and water. The *Chd8* V986* allele was generated in C57BL/6J blastocysts using CRISPR/Cas9 insertional mutagenesis by the UNC Animal Model Core facility. The insertion introduced tandem stop codons at amino acid positions V986 and E987 and a *MboI* restriction site. The male founder was backcrossed to C57BL/6J mice to eliminate potential unlinked off-target mutations. Genomic DNA was isolated from tail clips using Proteinase K digestion. Genotyping was performed by PCR amplification of genomic DNA with primers: (F) 5′ GCTAAGACAGAAATCTGATCTATTACCAGTAGA and (R) 5′ GGTCTTGAGATCCCCAAAATCCTTAA followed by *MboI* restriction enzyme digestion, to distinguish wild-type (WT; 227-bp product) and mutant (149-bp and 78-bp products) alleles. Animal protocols in this study were approved by the Institutional Animal Care and Use Committee at the University of North Carolina at Chapel Hill.

### Western blot

Brains were dissected from WT and *Chd8*^V986*/+^ mice and placed in radioimmunoprecipitation assay buffer (Sigma, R0278) with 1× phosphatase inhibitor cocktail (Sigma, P5726) and protease inhibitor cocktail (Sigma, P8340). Following sonication on ice for 2 × 15 s, lysates were centrifuged at 10,000×*g* at 4 °C for 20 min. Total protein was quantified in the supernatant using Bio-Rad Protein Assay Dye Reagent (Bio-Rad, 5000006). Protein (40 μg) was separated on a 4–20% pre-cast SDS/PAGE gel (Bio-Rad, 4568094) and transferred to an Immun-Blot PVDF membrane (Bio-Rad, 1620174), previously activated with methanol. The membrane was incubated with a blocking buffer (LI-COR, P/N: 927-70001) followed by incubation with rabbit anti-CHD8 (Abcam, ab84527; 1:1000) and mouse anti-alphaactin primary antibodies (Sigma-Aldrich, MAB1501; 1:1000) in blocking solution overnight at 4 °C. The membrane was washed with Tris-buffered saline with Tween 20 (TBST; 100 mM Tris pH 7.5, 165 mM NaCl, 0.1% v/v Tween 20) and incubated with secondary antibodies at a dilution of 1:10,000 [LI-COR, IRDye 680RD-congujated donkey anti-mouse polyclonal antibody (LI-COR, c6116-05) or 1:10,000 IRDye 800CW-conjugated donkey anti-rabbit (LI-COR, c60712-05)] in blocking buffer at room temperature for 2 h. The membranes were washed with TBST and imaged using a LI-COR Odyssey system.

### Behavior assessments

Subjects were *n* = 8 WT and *n* = 8 *Chd8*^V986*/+^ male mice and were tested in this order (age in weeks): elevated plus maze (26 weeks), open-field (27 weeks), social approach in a three-chamber choice task (28 weeks), marble burying assay and acoustic startle test (29 weeks), buried food test for olfactory ability (30 weeks), open-field (53 weeks), and social approach in a three-chamber choice task (55 weeks).

### Elevated plus maze

This test was used to assess anxiety-like behavior, based on a natural tendency of mice to actively explore a new environment versus a fear of being in an open area. Mice were given one 5-min trial on the plus maze, which had two walled arms (the closed arms, 20 cm in height) and two open arms. The maze was elevated 50 cm from the floor, and the arms were 30-cm long. Mice were placed on the center section (8 cm × 8 cm) and allowed to freely explore the maze. Measures were taken of time on and the number of entries into the open and closed arms.

### Open-field test

Exploratory activity in a novel environment was assessed by a 1-h trial in an open-field chamber (41 cm × 41 cm × 30 cm) crossed by a grid of photobeams (VersaMax system, AccuScan Instruments). Counts were taken of the number of photobeams broken during the trial in 5-min intervals, with measures taken of locomotion (total distance traveled), rearing movements, and time spent in the center region of the open field, an index of anxiety-like behavior.

### Three-chamber choice task

Mice were evaluated for social preference in a three-chamber choice task. The social testing apparatus was a rectangular, three-chambered box fabricated from clear Plexiglas. Dividing walls had doorways allowing access into each chamber. An automated image tracking system (Noldus Ethovision) provided measures of time spent in 5 cm proximity to each cage and numbers of entries into each side of the social test box. The procedure consisted of three 10-min phases: a habituation period, a test for sociability, and a test for social novelty preference. During habituation, the mouse was allowed to explore the chamber for 10 min. For the sociability assay, mice were given a choice between being in the proximity of an unfamiliar conspecific (stranger 1) versus an empty cage. The C57BL/6J adult male (stranger 1) mouse was enclosed in a small Plexiglas cage drilled with holes. An identical empty Plexiglas cage was placed in the opposite side of the chamber. The test mouse was allowed to explore the entire social test box for a 10-min session. In the social novelty phase, mice were given a choice between the already-investigated stranger 1 versus a new unfamiliar mouse (stranger 2). The test mouse was given an additional 10 min to explore the social test box.

### Tube co-occupancy test (TCOT)

A separate set of mice (age 8–10 weeks, 12 WT and 10 *Chd8*^V986*/+^, with equal numbers of males and females of each genotype) was evaluated in the TCOT, performed as previously described [29]. Briefly, two same-sex stranger mice (dyads) were placed at the same time into an arena with opaque Plexiglas walls (39-cm wide × 26-cm long × 12-cm high). The arenas were situated on top of a glass shelf 105 cm above the ground to create a visual cliff and were brightly illuminated with a 250-W LED light (∼ 3,000 lux). Each open-field box contained a single opaque PVC cylinder (7.5-cm long × 3 cm in diameter or placed against one long wall. Dyads were tested for 1 h, after a 1-h habituation period to the TCOT arena. Stranger mice were born of different parents with no contact before testing. All animals were age, genotype, and sex-matched and were tested only once in the TCOT. A digital video camera was placed directly over the arena. Scoring occurred manually, by observing one 10-s sample every 2 min, generating percentages of samples featuring tube co-occupancy, single occupancy, or vacancy.

### Marble-burying assay for exploratory digging

Mice were tested in a Plexiglas cage located in a sound-attenuating chamber with ceiling light and fan. The cage contained 5 cm of corncob bedding with 20 black glass marbles (14-mm diameter) arranged in an equidistant 5 × 4 grid on top of the bedding. Subjects were given access to the marbles for 30 min. Measures were taken of the number of buried marbles (two thirds of the marble covered by the bedding).

### Acoustic startle test

This procedure was used to assess auditory function, reactivity to environmental stimuli, and sensorimotor gating. The test was based on the reflexive whole-body flinch, or startle response, that follows exposure to a sudden noise. Measures were taken of startle magnitude and prepulse inhibition, which occurs when a weak prestimulus leads to a reduced startle in response to a subsequent louder noise. Mice were placed into individual small Plexiglas cylinders within larger, sound-attenuating chambers. Each cylinder was seated upon a piezoelectric transducer, which allowed vibrations to be quantified and displayed on a computer (San Diego Instruments SR-Lab system). The chambers included a ceiling light, fan, and a loudspeaker for the acoustic stimuli. Background sound levels (70 dB) and calibration of the acoustic stimuli were confirmed with a digital sound level meter (San Diego Instruments). Each session consisted of 42 trials that began with a 5-min habituation period. There were seven different types of trials: the no-stimulus trials, trials with the acoustic startle stimulus (40 ms; 120 dB) alone, and trials in which a prepulse stimulus (20 ms; either 74, 78, 82, 86, or 90 dB) occurred 100 ms before the onset of the startle stimulus. Measures were taken of the startle amplitude for each trial across a 65-ms sampling window, and an overall analysis was performed for each subject’s data for levels of prepulse inhibition at each prepulse sound level (calculated as 100 - [(response amplitude for prepulse stimulus and startle stimulus together/response amplitude for startle stimulus alone) × 100].

### Buried food test for olfactory function

Several days before the olfactory test, an unfamiliar food (Froot Loops, Kellogg Co., Battle Creek, MI) was placed overnight in the home cages of the mice. Observations of consumption were taken to ensure that the novel food was palatable. Sixteen to 20 h before the test, all food was removed from the home cage. On the day of the test, each mouse was placed in a large, clean tub cage (46 cm L × 23.5 cm W × 20 cm H), containing paper chip bedding (3-cm deep), and allowed to explore for 5 min. The mouse was removed from the cage, and one Froot Loop was buried in the cage bedding. The mouse was then returned to the cage and given 15 min to locate the buried food. Measures were taken of latency to find the food reward.

### Immunohistochemistry

Mice were perfused with 4% paraformaldehyde in 0.1 M phosphate buffer, pH 7.4. The brains were dissected and immersion fixed in the same fixative buffer, freshly prepared, for 24 h, followed by cryoprotection in 30% sucrose in 0.1 M phosphate buffer, pH 7.4 at 4 °C. Each brain was cut on a cryostat into 60-μm sections and held at − 20 °C in a solution containing glycerol (25% v/v), ethylene glycol (30% v/v), and phosphate-buffered saline (PBS, pH 7.4; 45% v/v) for immunostaining at a later date. Sections were removed from this storage solution and sequentially rinsed with PBS and a Tris-buffered saline containing Triton-X (TBS/TX: 0.05 M Tris hydroxymethyl aminomethane, 2.7% NaCl, 0.3% Triton-X-100; pH 7.6) before blocking in 10% normal donkey serum (NDS; EMD-Millipore, S30-100ML) in TBS/TX and treatment with primary antibodies diluted in 10% NDS/TBS/TX overnight at room temperature. The following day, sections were once again rinsed in TBS/TX and blocked with 10% NDS/TBS/TX before application of a secondary antibody cocktail diluted in NDS/TBS/TX to which DAPI (ThermoFisher, EN62248) was added for a final concentration of 1:4,000. After a 6 h incubation in secondary antibodies, sections were rinsed in PBS and treated with TrueBlack Lipofuscin Autofluorescence Quencher (Biotium, 23007) diluted in 70% ethanol for 2 minutes, rinsed again in PBS, floated onto SuperFrost Plus slides (Fisher Scientific, 12-550-15), briefly dried, and coverslipped with Fluoro Gel mounting medium (Electron Microscopy Sciences, 17985-10). We used antibodies to Phospho-S6 Ribosomal Protein (Ser235/236) (Cell Signaling, 4857; 1:100), a rabbit monoclonal, and NeuN (EMD-Millipore, ABN90; 1:400), a guinea pig polyclonal. Secondary antibodies (donkey anti-rabbit Alexa Fluor 488, A-21206, and donkey anti-guinea pig Alexa Fluor 647, 706-605-148) were purchased from Invitrogen and Jackson ImmunoResearch, respectively, and used at 1:200. Sections were imaged on a Zeiss LSM 710 confocal microscope.

### RNA extraction

Mice were sacrificed at E14.5, and at 1, 6, and 12 months of age. The brains were removed, and the cerebral cortex (one hemisphere) was dissected and stored at − 80 °C until RNA extraction. Six replicates were prepared for both WT and *Chd8*^V986*/+^ at all four time points for a total of 48 samples. All samples were processed in parallel, including meticulous planning to ensure that equal numbers of each genotype and time point were centrifuged together, to minimize batch effects associated with any part of processing, handling, RNA isolation, or RNA-seq library preparation. To lyse the samples, 350 μl of Qiagen RLT Plus buffer (with 20 μL DTT per mL buffer) per 20-mg tissue was added. A hand-held motorized homogenizer was used to immediately homogenize that samples. The lysate (350 μL) was processed through a RNeasy Plus Mini Kit for RNA extraction following the manufacturer protocol (Qiagen, 74136). RNA concentration was measured using Qubit RNA Broad-Range Assay and stored at − 80 °C (Thermo Fisher Scientific, Q10211).

### RNA sequencing

PolyA RNA-stranded libraries were prepared (KAPA, Roche), and sequencing was performed (Illumina) at the UNC High-Throughput Sequencing Facility. To minimize sequencing related batch effects, each replicate was barcoded (KAPA, Roche) and multiplexed such that all samples were represented on a given lane of a flowcell, then each library was sequenced on a HiSeq 4000 with stranded paired end 50 bp reads. Reads were filtered for a quality score of 20 or more in at least 90% of all bases using fastq_quality_filter in the FASTX toolkit 0.0.14 (http://hannonlab.cshl.edu/fastx_toolkit/index.html). Sequencing adapters were trimmed using cutadapt 1.12 [30], and reads were then aligned to the mm9 reference genome using STAR 2.5.2b [31]. Read depth (passing reads that aligned to the genome) for each sample can be found in Supplementary Data File 1. Transcripts were quantified using Salmon 0.11.3 [32], and differential expression was detected using DESeq2 1.22.2 [33], using a model that corrected for batch effects and a threshold of adjp < 0.1. Sequencing data are available at GEO under accession GSE142208. Differentially expressed genes from other studies of *Chd8* transgenic heterozygotes that provided RNA-seq analysis results [16, 17, 19, 20] were compared for overlap of significant upregulation and downregulation of transcription. Overlaps were visualized using the UpSetR package in R [34]. The significance of pairwise overlaps of the upregulated and downregulated genes, by time point, in the present study and in the other previously mentioned studies was tested using the web interface for gprofiler (https://biit.cs.ut.ee/gprofiler/gost) [35]. A custom GMT file was used consisting of the default gprofiler GMT, with entries added for the upregulated and downregulated gene lists from the previously mentioned *Chd8* transgenic studies. Overlaps were considered significant with an adjusted *p* value < 0.05.

### Hierarchical clustering analysis

We first created a union set of differentially expressed genes from all four ages. Fold-changes were standardized by dividing the fold-change by the maximum absolute fold-change value for that gene across all four time points, thus constraining the values to a range of − 1 to 1. Genes were then clustered hierarchically using 1– Pearson correlation distance. The resulting tree was cut at a height of 1.6, resulting in 7 clusters of genes. The genes were then filtered to remove those with Pearson correlation < 0.5. Clusters with fewer than 30 genes remaining after filtering were excluded from further analysis, resulting in a final set of five gene clusters.

### Pathway analysis

To determine which pathways were enriched among the five clusters, we used gprofiler 0.1.6 [35] and ran it in R 3.5.3 [36]. Pathways were considered significant at adjusted *p* value < 0.05. The variance-stabilizing transformation (VST)-normalized expression values for each genotype underlying these fold-changes were then aggregated across all significant genes in each pathway by taking the average on a replicate by replicate basis.

### Statistical analysis

For each behavior procedure, measures were taken by an observer blind to mouse genotype. Data were analyzed using one-way or repeated measures analysis of variance (ANOVA). Fisher’s protected least-significant difference tests were used for comparing group means only when a significant *F* value was determined. Within-genotype ANOVAs were used to determine side preference in the three-chamber test. Data presented in figures throughout the study represent means (± SEM). For all comparisons, significance was set at *p* < 0.05. Significant *p* values and group sizes are reported in figure legends. Sample *n* in reported behavioral experiments was appropriate assuming a large effect size (*f* = 0.8) for 4 repeated measures between 2 groups (WT versus *Chd8*^V986*/+^).

## Results

### Generation of *Chd8*^V986*/+^ mice

**Fig. 1:**
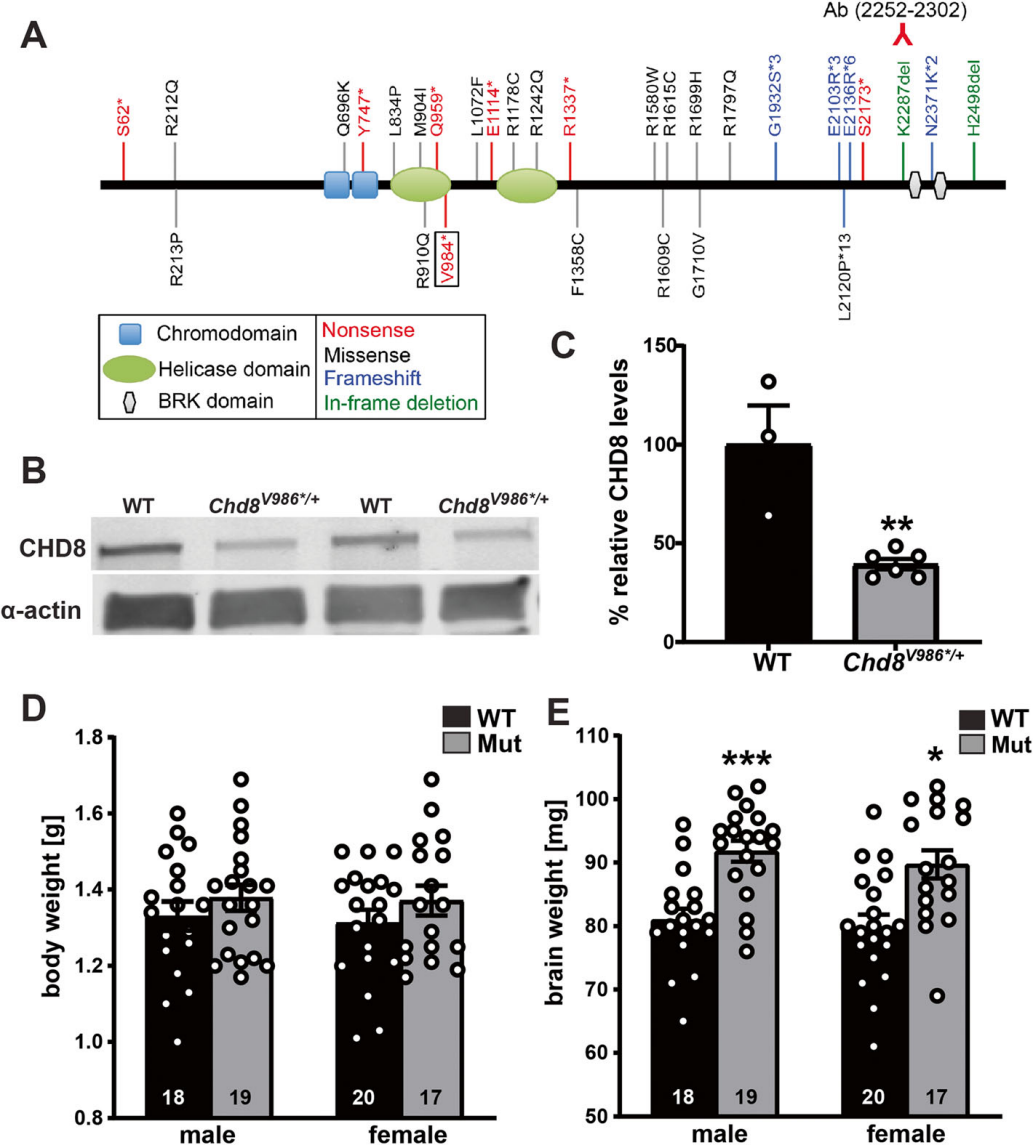
Increased brain weight at birth in *Chd8*^V986*/+^ haploinsufficient mice*.* **a** ASD-linked mutations in human CHD8. The mouse CHD V986* mutation is synonymous with human CHD8 V984* (boxed). The region recognized by the CHD8 antibody (Ab) is shown. **b** Western blot and **c** quantification of CHD8 protein (290 kDa) in brain lysates from WT and *Chd8*^V986*/+^ mice; age P0. Data represents mean of 3–6 samples per genotype ± S.E.M., **P < 0.01. WT: wild-type; Mut: *Chd8*^V986*/+^*.* **d**, **e** Body and brain weights of WT and *Chd8*^V986*/+^ mice at P0. Data are mean of 17–20 samples per genotype ± S.E.M. *P < 0.05; ***P < 0.001

ASD-linked indels, missense, and nonsense mutations are found throughout the protein-coding region of the human *CHD8* gene (Fig. 1a). Several of these mutations introduce premature stop codons that presumably result in null alleles via nonsense-mediated decay (loss-of-function). We used CRISPR/Cas9 technology to introduce the valine 984 stop codon [9] at the equivalent position in mouse *Chd8* (V986*) on a pure C57BL/6J background. Homozygous *Chd8*^V986*/V986*^ pups were never recovered in P0 litters from *Chd8*^V986*/+^ × *Chd8*^V986*/+^ (heterozygous × heterozygous) mating pairs, consistent with this mutation being an embryonic lethal null allele [37]. CHD8 protein levels were reduced by half in the brain of *Chd8*^V986*/+^ mice (Fig. 1b, c). *Chd8*^V986*/+^ male and female mice were comparable in body weight at birth (P0) but displayed significantly increased brain weight when compared to WT littermate controls (Fig. 1d, e). At later ages, the body weight of *Chd8*^V986*/+^ males was significantly lower than WT controls (25 weeks: 32.8 ± 0.52 g versus 29.9 ± 0.50 g).

### Pup survival is reduced when raised by *Chd8*^V986*/+^ dams

**Fig. 2:**
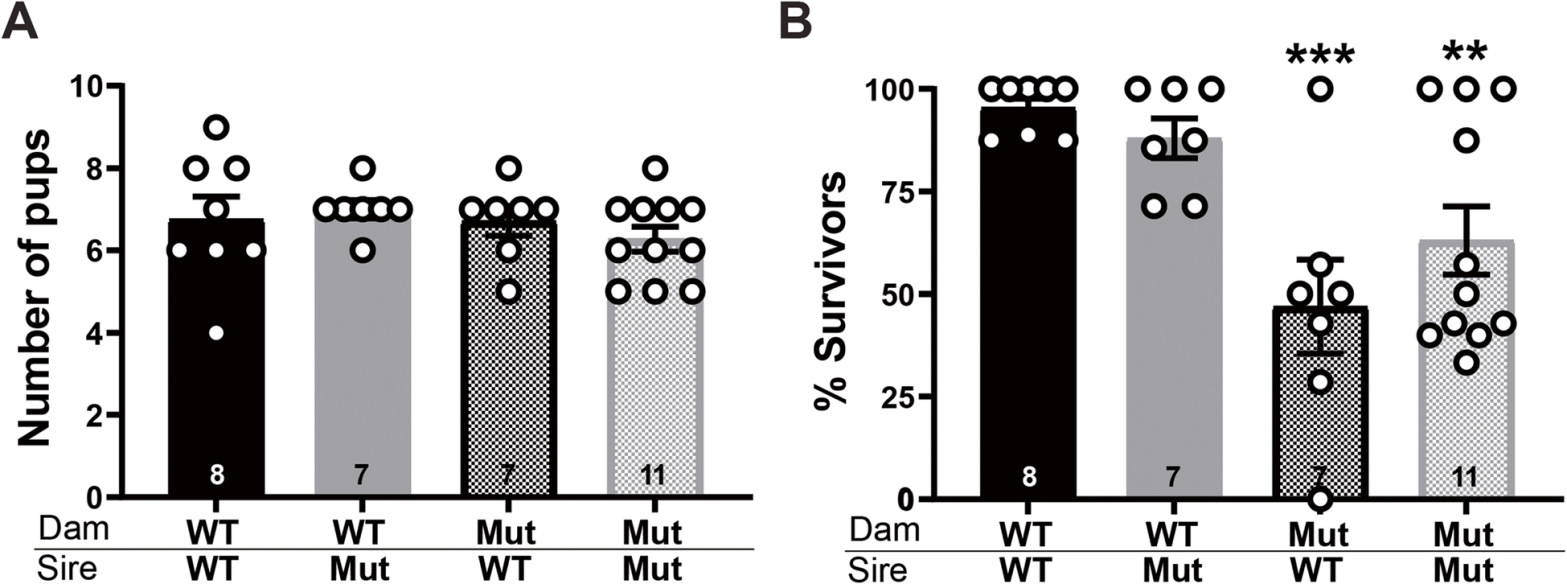
Reduced pup survival when reared by *Chd8*^V986*/+^ dams. **a** Litter size at birth (age=P0). No significant differences. **b** Pup survival at P2 relative to the number of pups present at P0. Genotype of the dam and the sire is indicated. Data represents mean from 8–11 l (*n* indicated in bars) ± S.E.M., **p < 0.01, ***p < 0.001 relative to WT × WT cross

We also evaluated whether the genotype of the mother or father affected pup survival. Maternal or paternal genotype did not have an effect on the number of offspring at P0 (Fig. 2). However, pup survival at P2 was significantly reduced only when litters were reared by *Chd8*^V986*/+^ dams (Fig. 2).

### Behavioral abnormalities in the open-field test worsen with age in *Chd8*^V986*/+^ mice

**Fig. 3:**
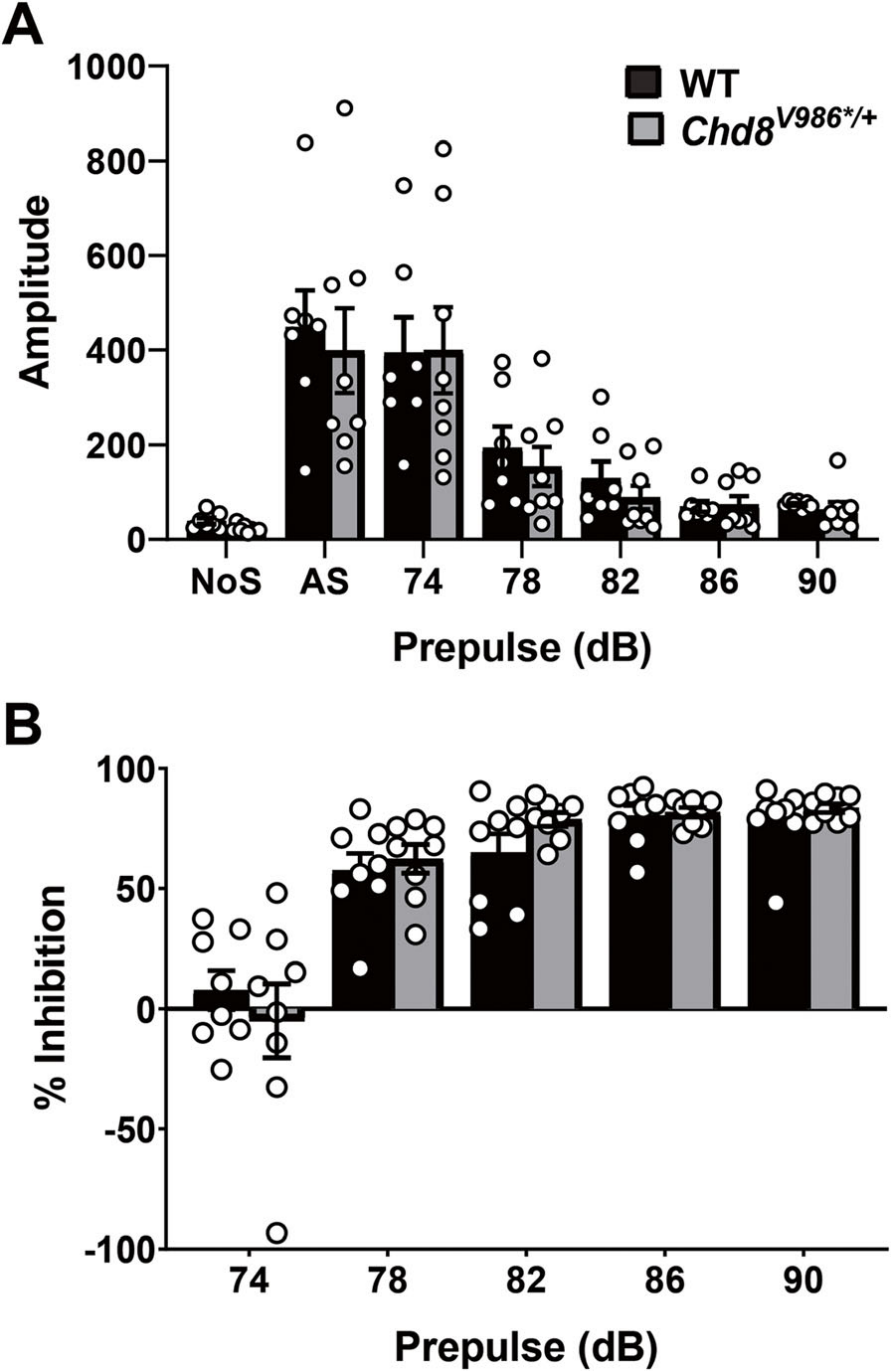
Acoustic startle response. **a** Magnitude of startle responses and **b** prepulse inhibition following presentation of acoustic stimuli. Trials included no stimulus (NoS) trials and acoustic startle stimulus (120 dB) alone trials. No significant differences between genotypes

ASD disproportionally affects males over females (~ 4:1), ASD-linked mutations in *CHD8* are more common in males (3.5:1) [13] and only *Chd8* mutant male mice (with a different heterozygous loss-of-function allele, Asn2371LysfsX2; shown in Fig. 1) had behavioral abnormalities [20]. These data suggest that the penetrance of loss-of-function mutations in *Chd8* is influenced by sex, with penetrance higher in males. Thus, we next evaluated a cohort of male WT and *Chd8*^V986*/+^ mice in a battery of behavioral tests (see the “Methods” section). At 6 months of age, WT and *Chd8*^V986*/+^ mice spent a similar percentage of time on the open arms of the elevated plus maze and had a similar number of total entries during the test (Table 1). Moreover, WT and *Chd8*^V986*/+^ mice had similar performance in the marble-burying test (Table 1), found buried food with similar latencies (Table 1), showed a comparable magnitude of startle responses (Fig. 3), and demonstrated similar prepulse inhibition in the acoustic startle test (Fig. 3).

**Table 1:** Tests for anxiety-like behavior, perseverative responses, and olfactory function

	WT	*Chd8*^V986*/+^
Elevated plus maze		
Percent open-arm time	21 ± 2	20 ± 2
Percent open-arm entries	30 ± 3	34 ± 3
Total number of entries	24 ± 2	21 ± 2
Marble-bury assay		
Number of marbles buried	17 ± 0.7	15 ± 1.4
Olfactory test		
Latency to find buried food(s)	36 ± 9	41 ± 13

No significant differences between genotypes

**Fig. 4:**
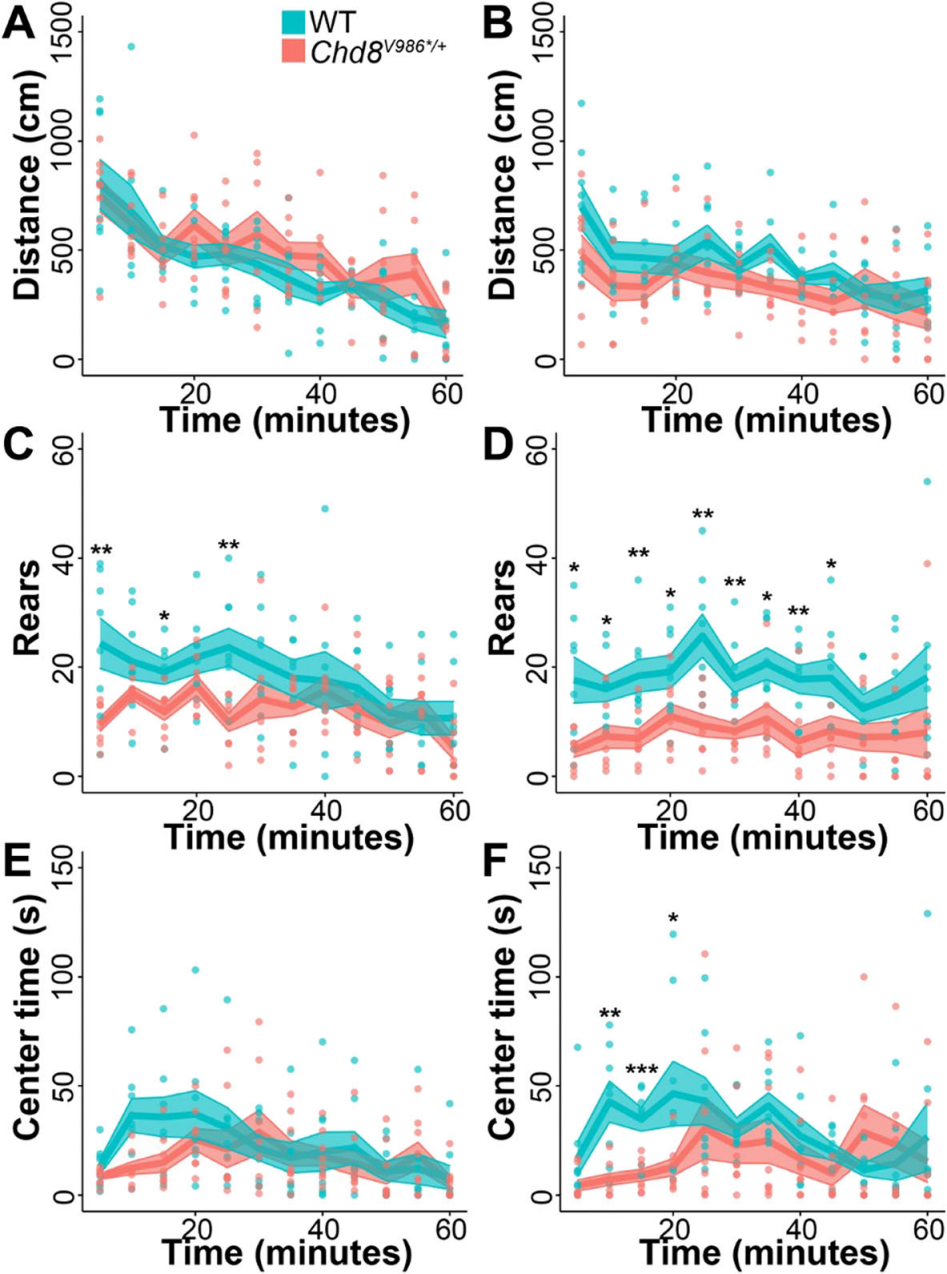
Open-field behaviors as a function of age. WT and *Chd8*^V986*/+^ male mice were tested at (**a**, **c**, and **e**) 6 months of age and at (**b**, **d**, **f**) 12 months of age. **a**, **b** Distance traveled. No significant differences. **c**, **d** Number of rears. Six-month-old [genotype × time interaction, *F*(11,154) = 2.36, *p* = 0.01] and 1-year-old [main effect of genotype, *F*(1,14) = 10.0, *P* = 0.0069] *Chd8*^V986*/+^ mice showed a significant decrease in rearing movements relative to age-matched WT controls. **e**, **f** Time in center. No significant differences between 6-month-old WT and *Chd8*^V986*/+^ mice [genotype × time interaction, *F*(11,154) = 1.83, *p* = 0.0536]. Twelve-month-old *Chd8*^V986*/+^ mice showed a significant reduction in center time behavior relative to age-matched WT controls [genotype × time interaction, *F*(11,154) = 2.91, *p* = 0.0016]. Lines and shaded areas represent means ± S.E.M. Each data point represents an individual mouse. *p < 0.05, **p < 0.01, ***p < 0.001

In contrast, phenotypes were detected in the open-field test and in social tests at 6 months of age, so we repeated these tests in the same cohort at 1 year of age to assess reproducibility and severity as a function of age. We found that WT and *Chd8*^V986*/+^ mice had comparable locomotor activity in the open field test at 6 months and 12 months of age (Fig. 4a, b); however, *Chd8*^V986*/+^ mice showed a significant decrease in rearing movements at 6 months [genotype × time interaction, *F*(11,154) = 2.36, *p* = 0.01] (Fig. 4c). By 12 months, this rearing deficit was much more evident [main effect of genotype, *F*(1,14) = 10.0, *p* = 0.0069] (Fig. 4d).

A similar pattern of age-dependent genotype differences was observed for the measure of time spent in the center region. At 6 months of age (Fig. 4e), *Chd8*^V986*/+^ mice demonstrated a non-significant trend for decreased time spent in the center [genotype × time interaction, *F*(11,154) = 1.83, *p* = 0.0536]. By 12 months of age (Fig. 4f), the differences between the genotypes in center time was highly significant [genotype × time interaction, *F*(11,154) = 2.91, *p* = 0.0016].

### Increased social interactions and social novelty preference of *Chd8*^V986*/+^ mice

**Fig. 5:**
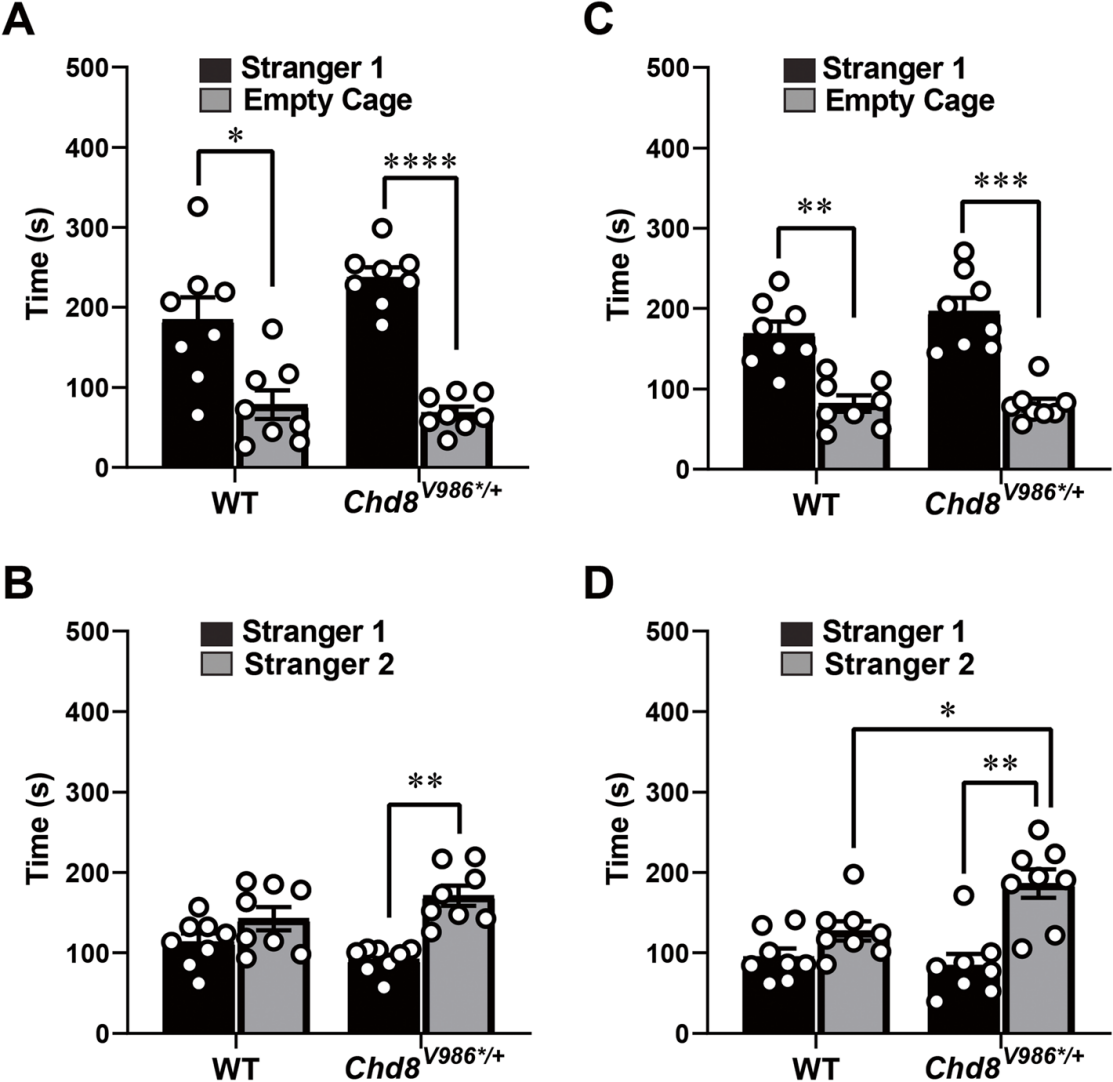
Three-chamber social behavior tests as a function of age. WT and *Chd8*^V986*/+^ male mice were tested at (**a**, **c**) 6 months of age and (**b**, **d**) 12 months of age. **a**, **b** Time spent in 5 cm proximity to a caged stranger or an empty cage during the sociability test. **c**, **d** Time spent in 5 cm proximity to each caged stranger mouse during the social novelty preference test. Bars represent mean ± S.E.M. for a 10-min test. *p < 0.05, **p < 0.01, ***p < 0.001, ****p < 0.0001

At 6 and 12 months of age, WT and *Chd8*^V986*/+^ mice preferred to spend more time in proximity to the cage containing the stranger mouse versus the empty cage [within-genotype comparisons following repeated measures ANOVA, a significant effect of side, test 1, *F*(1,14) = 36.29, *p* < 0.0001; test 2, *F*(1,14) = 51.47, *p* < 0.0001] (Fig. 5a, b). However, at 6 months of age (Fig. 5c), only the *Chd8*^V986*/+^ mice exhibited a shift in preference to the newly introduced stranger 2 [within-genotype comparisons following repeated measures ANOVA, a significant effect of side, *F*(1,14) = 17.43, *p*=0.0009]. This genotype-dependent social novelty preference was more evident by 12 months of age (Fig. 5d), during which the *Chd8*^V986*/+^ mice spent significantly more time in proximity to stranger 2 than WT mice [main effect of genotype, *F*(1,14) = 5.39, *p* = 0.0359; and side, *F*(1,14) = 16.49, *p* = 0.0012]. Consistent with these findings, TCOT behavior suggests increased social interest between 8 and 10-week-old *Chd8*^V986*/+^ mouse dyads relative to WT mouse dyads (Supplementary Figure S1). This increased social preference was not due to increased aggression, since fighting was observed in less than 3% of samples during testing.

### Transcriptomic changes in the brain as a function of age

We next used RNA-seq to evaluate gene expression in the cerebral cortex of WT and *Chd8*^V986*/+^ male mice at embryonic (E14.5) and postnatal ages (1, 6, and 12 months). All samples were processed and sequenced in parallel to minimize batch effects and to enable comparisons across genotypes and ages. *Chd8* expression was highest at E14.5 and persisted at a lower level throughout life (Supplementary Figure S2), consistent with Xu et al. [38]. *Chd8* expression was significantly reduced at each time point in *Chd8*^V986*/+^ mice (Supplementary Figure S2). Evaluation of reads spanning the WT V986 and mutant V986* allele indicated that the mutant allele was not expressed, suggestive of nonsense-mediated decay. Two other genes aside from *Chd8* were differentially expressed across all four time points (*Usp11*, *Wars2*), and 11 genes were differentially expressed in three of the four time points (*Asl*, *BC025920*, *Crlf2*, *Csad*, *Ddo*, *Egfl6*, *Lyrm7*, *Parva*, *Pum3*, *Tmem209*, *Zbtb45*). There thus appears to be a core set of genes that are reproducibly affected in the brain of *Chd8*^V986*/+^ mice irrespective of age.

After identifying differentially expressed genes at each age (Supplementary Data File 2), we hierarchically clustered standardized log_2_-fold change values of these genes as a function of age. This standardization approach enabled us to identify genes with similar temporal changes in expression. This analysis revealed five gene clusters that temporally differed between WT and *Chd8*^V986*/+^ samples. The full lists of genes in each cluster are found in Supplementary Data File 3.

**Fig. 6:**
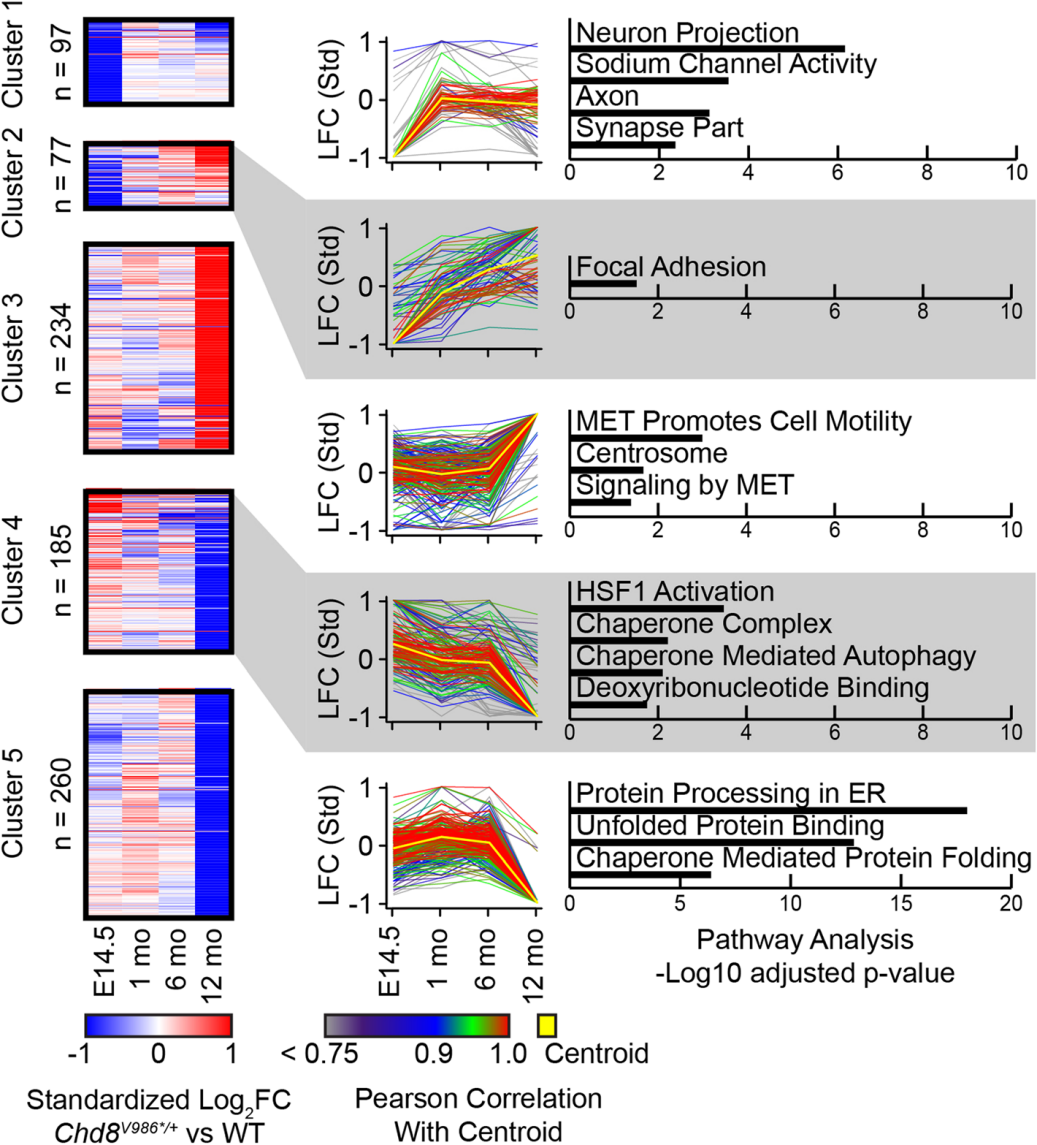
Clustering of differentially expressed genes by temporal patterns. (Left) Heatmaps show clustering of the union set of differentially expressed genes (*n* = 853 total), plotting standardized log_2_ fold-changes. (Middle) Line plots show the change in log_2_ fold-change over time for all genes in the cluster, colored by Pearson correlation to the centroid of the cluster. (Right) Barplots of the − log_10_ adjusted *p* value of selected pathways significantly associated with genes in each cluster

Gene ontology/pathway analyses were then performed with these cluster-specific genes (Fig. 6, Supplementary Data File 4). At E14.5, genes associated with sodium channel activity and synaptic function were reduced in *Chd8*^V986*/+^ samples relative to WT controls, implying delayed neuronal maturation and/or abnormal neuronal communication (cluster 1; Fig. 6, Supplementary Figure S3). Expression of these genes equalized to that of WT at later ages (cluster 1; Fig. 6, Supplementary Figure S3). Genes associated with focal adhesion were also reduced in *Chd8*^V986*/+^ cortical samples at E14.5 and then increased relative to WT samples at 12 months of age (cluster 2; Fig. 6, Supplementary Figure S3). These gene expression changes associated with clusters 1 and 2, combined with macrocephaly at birth, support a neurodevelopmental abnormality in *Chd8*^V986*/+^ mice.

The largest number of differentially expressed genes was associated with clusters 3, 4, and 5, which showed pronounced changes in 12-month-old mice. Pathways associated with the unfolded protein response (UPR), endoplasmic reticulum (ER) stress, and chaperone-mediated protein folding were reduced in *Chd8*^V986*/+^ cortical samples relative to WT samples at 12 months of age (cluster 5, Fig. 6), all suggestive of impaired proteostasis and/or a blunted response to misfolded proteins.

**Fig. 7:**
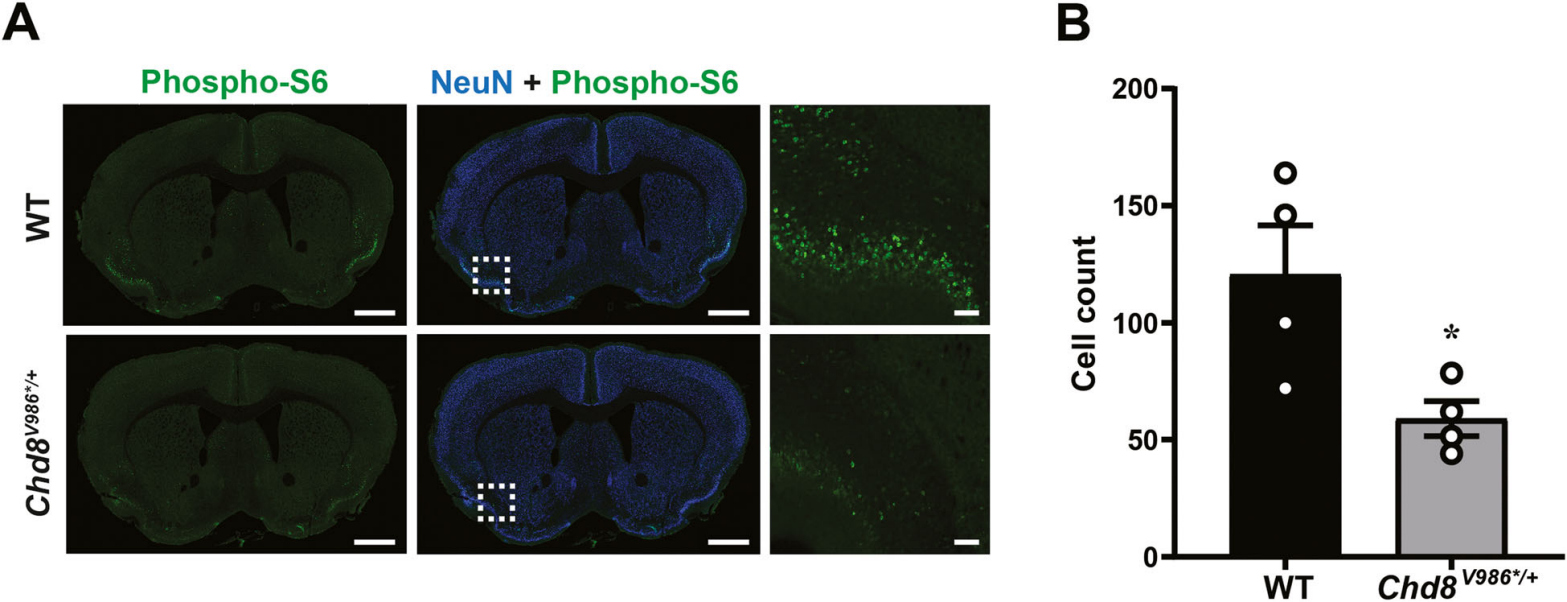
Reduced phospho-S6 immunostaining in the piriform cortex of aged *Chd8*^V986*/+^ mice. **a** Immunohistochemistry of NeuN (blue) and phospho-S6 (green) in 12-month-old mice and **b** quantification of phospho-S6-positive cells. Each data point indicates the average of two 500 × 500 pixel representative regions of the piriform cortex ± S.E.M. with 4 male mice per genotype. Scale bar (left and middle panels) = 1 mm, (right panels) = 100 μm. *p < 0.05

ER stress triggers the activation of the mammalian target of rapamycin complex 1 (mTORC1), which can be monitored by phosphorylation of ribosomal protein S6 (phospho-S6) [39–41]. We then stained for phospho-S6 in brain sections from 12-month-old WT and *Chd8*^V986*/+^ mice. Phospho-S6 staining was significantly reduced, particularly in piriform cortex (Fig. 7), consistent with reduced ER stress in *Chd8*^V986*/+^ cortical samples. mTORC1 also activates the inositol-requiring enzyme 1 (IRE1) signaling pathway that cleaves X-box binding protein 1 (XBP1) mRNA, which translocates to the nucleus to upregulate target genes. Aged *Chd8*^V986*/+^ mice showed decreased XBP1 expression (cluster 5, Fig. 6, Supplementary Figure S4), consistent with reduced mTORC1 and IRE1 pathway activation.

Proteostasis is also maintained in the cytosol and nucleus via activation of the heat shock response pathway [42]. We found that genes associated with heat shock factor 1 (HSF1) signaling and chaperone function were reduced in 12-month-old *Chd8*^V986*/+^ samples (cluster 4, Fig. 6). Additionally, genes associated with c-MET signaling pathways were upregulated in *Chd8*^V986*/+^ samples (cluster 3, Fig. 6), as would be expected if the ER stress response was reduced [43, 44]. Collectively, our data suggest that protein homeostasis is impaired in aged *Chd8*^V986*/+^ brain samples. Our approach to study phenotypes longitudinally across the first year of life uncovered distinct molecular pathways that are abnormal in the brain of embryonic and older adult *Chd8*^V986*/+^ mice.

## Discussion

We generated a new *Chd8* mutant mouse line with 50% less CHD8 protein in the brain, allowing us to evaluate behavioral and brain transcriptomic consequences of *Chd8* haploinsufficiency throughout life. Our data reproduce and extend a role for CHD8 in brain development [15, 16, 19, 20] and further indicate that macrocephaly at birth is a common phenotype in almost all *Chd8* mutant mouse lines (Table 2). Likewise, macrocephaly is commonly observed in humans carrying disruptive *CHD8* mutations [10, 14, 21]. By evaluating age as a variable, we also found that genes associated with UPR/ER stress were reduced exclusively in aged *Chd8*^V986*/+^ mice. These data suggest that *Chd8* haploinsufficiency impairs proteostasis later in life and/or that normal age-related responses to misfolded proteins are blunted in *Chd8*^V986*/+^ mice [45]. While transcriptomic analyses in post-mortem human brain samples revealed signs of neuroimmune activation in about half of all individuals with ASD [25, 26], our data does not suggest an increase in immune-related gene expression in aged *Chd8*^V986*/+^ mice. *Chd8* haploinsufficiency may simply not trigger neuroimmune activation at any age. Alternatively, it may be necessary to examine *Chd8*^V986*/+^ mice beyond 1 year of age, or it may be necessary to couple *Chd8* haploinsufficiency with an immune-targeting environmental insult in the prenatal period [46, 47].

**Table 2:** Summary of phenotypes in *Chd8* haploinsufficient mouse lines

	This study	Platt et al.	Katayama et al.	Gompers et al.	Suetterlin et al.	Jung et al.
Age range tested (weeks)	25–52	10–14	13–17	8–16	9–12	8–14
Body weight	Decrease (25 weeks)	Decrease (10 weeks)	No change (9 weeks)	No change	Decrease (5 weeks)	No change (3 weeks)
Brain weight or volume at birth	Increase	X	Increase	Increase	Increase	X
Pup survival, reared by *Chd8* mutant dams	Reduced	X	X	X	X	X
Elevated plus maze						
% open arm time	No change	X	Decrease	X	X	No change
Open field						
Total distance	No change	Decrease	No change	No change	Decrease	Decrease
Rearing	Decrease	X	X	X	X	X
Center time	Decrease	Decrease	Decrease	X	No change	Decrease
Three chamber test						
Sociability	No change	No change	No change	No change	Increase	No change
Novelty preference	Increase	Decrease	Decrease	X	X	No change
Entries	No change	No change	X	No change	X	X
Marble-bury assay	No change	No change	X	No change	Decrease	No change
Acoustic startle						
Amplitude	No change	X	Decrease	X	X	X
Prepulse inhibition	No change	X	Increase	X	X	No change
Olfactory test	No change	X	X	X	X	No change

*X* indicates not conducted or not reported. Consult the indicated references for details as methodological differences may affect or limit comparisons between studies

Intriguingly, we found that very few differentially expressed genes were shared across other *Chd8* mouse models, even when comparing similar ages (Supplementary Figure S5, Supplementary Data File 5). *Chd8* was significantly downregulated in more studies than any other gene (Supplementary Figure S5A, column 102). *Chd8* was not significantly downregulated in the Gompers E14.5 time point and in all of the Katayama study time points (curiously, *Chd8* was significantly upregulated in the Katayama E10.5 and E12.5 time points (Supplementary Figure S5A, column 10)). There was limited statistically significant overlap between the differentially expressed genes in this study and those from previous studies (Supplementary Figure S5C). The most significant overlaps were with the E14.5 upregulated genes in the Katayama and Suetterlin studies. We can only speculate as to why greater transcriptomic overlap with other studies was not observed. We rigorously controlled for batch effects and deeply sequenced six biological replicates per genotype per time point. As is well-known, batch effects, shallow sequencing depth, and small sample size can all negatively impact the statistical significance and reproducibility of RNA-seq-based analyses.

Although social deficits are most often associated with autism-like behavior phenotypes, there is growing evidence that hypersociability can also be observed in mouse models of neurodevelopmental disorders [48]. We found that *Chd8*^V986*/+^ mice spent greater time investigating a new stranger (Fig. 5). Additionally, *Chd8*^V986*/+^ mice show higher levels of tube co-occupancy compared to WT mice (Supplementary Figure S1), suggestive of greater interest in maintaining close physical proximity to other mice [29]. Other studies with *Chd8* mouse models reported higher levels of sociability in a three-chamber test [19] or increased duration of social contacts in tests of direct social interaction [16, 18, 19], although not all results have been consistent (Table 2). Inhibitory processes regulating the social approach may thus be disabled or impaired in *Chd8* mutant mice. We also found that pup survival was reduced when reared by *Chd8*^V986*/+^ dams. This phenotype has not previously been reported and suggests *Chd8* mutant dams are less capable of detecting and/or responding to social and non-social cues from the pups. The inferior nurturing ability of *Chd8* mutant dams could also be explained by low lactational yield and/or poor milk quality leading to malnourished pups. A direct evaluation of maternal behaviors is warranted in future studies. Since behavioral phenotypes worsened with age, our work indicates it might be possible to unmask phenotypes by using older mice with other loss-of-function mutations in *Chd8*, and possibly in mice with mutations in other high confidence ASD genes.

*Chd8*^V986*/+^ mice showed less rearing in the open field and spent less time in the center of the open field (Fig. 4). This behavioral difference in the open field could be explained by an increase in anxiety, as significant differences were observed shortly after introducing the mice to the testing chamber. Differences in behavior persisted after prolonged exposure to the open field, which may be suggestive of a reduction in exploration and locomotion. The elevated plus maze did not reveal any evidence for general anxiety phenotypes in *Chd8*^V986*/+^ mice (Table 1), further suggesting the interpretation of reduced activity and exploratory behavior from the open field. However, individuals with ASD often report increased anxiety, including patients with truncating mutations in CHD8 [9]. Nearly all *Chd8* haploinsufficient mouse lines studied to date show similar phenotypes in the open-field test (Table 2). Increased anxiety-like behaviors are also frequently detected in other mouse models of ASD [18, 49].

Homeostatic mechanisms maintain an appropriate level of neuronal activity despite ongoing challenges to neural networks [50]. These mechanisms are important for defining the mature constellation of ion channels and synaptic receptors that regulate neuronal function. Our RNA sequencing data shows that the embryonic (E14.5) *Chd8*^V986*/+^ cerebral cortex has altered expression of genes related to the maintenance of excitatory-inhibitory balance (Fig. 6, cluster 1), a common neurobiological feature of ASD. Moreover, embryonic brain knockdown of *Chd8* disrupted axon projections and delayed neuronal migration in mice and, like we found with synaptic gene expression (cluster 1), these phenotypes recovered shortly after birth [38]. Reduced synaptogenesis and/or axon development during the embryonic period has the potential to alter network function later in life [51].

Cell adhesion molecules play a critical role in synapse development and mutations in some of these genes increase the risk for ASD [52]. Our transcriptomic data suggest that genes associated with focal adhesion pathways are downregulated at E14.5, further suggesting that synaptogenesis or neuronal maturation may be impaired. A subset of these focal adhesion genes gradually increases above WT levels by 12 months (Fig. 6, cluster 2). The reason for this age-associated increase is unclear, but could compensate for the subset of synapse associated genes that are downregulated at the 12-month time point (Fig. 6, cluster 1).

The UPR upregulates three distinct transcription factors. The protein kinase-like ER kinase (PERK)-EIF2α pathway selectively induces activating transcription factor 4 (ATF4), thereby enhancing the expression of pro-apoptotic genes [39, 40]. ER stress also induces cleavage of activating transcription factor 6 (ATF6) which results in transcription of ER chaperones, including a 78-kDa glucose-regulated protein (GRP78) [39, 40]. Lastly, UPR induces activation of mTORC1, which can be monitored by phospho-S6 [39–41]. We found that expression of *Atf4* and the GRP78 gene *Hspa5* were reduced in 12-month-old *Chd8*^V986*/+^ mice (*Atf4* and *Hspa5* log_2_ fold-change = − 0.17 and − 0.61 and adjp = 0.069 and 0.019, respectively), and phospho-S6 levels were reduced in 12-month-old *Chd8*^V986*/+^ mice (Fig. 7), all consistent with reduced UPR. Moreover, mTORC1 activates the IRE1 signaling pathway that cleaves XBP1 mRNA, resulting in the induction of ER chaperones that reinforce ER folding capacity [39–41]. Expression of *Xbp1* was reduced in 12-month-old *Chd8*^V986*/+^ samples (log_2_ fold-change = − 0.35, adjp = 0.059), further suggesting UPR is reduced in aged *Chd8* mutants. It should be emphasized that the change in expression of *Xbp1* was trending toward significance, and the magnitude of this reduction was relatively small. Future studies are warranted to determine if UPR genes are reduced to a greater extent in *Chd8* mutants older than 1 year of age.

c-MET is the receptor for a hepatocyte growth factor (HGF), and c-MET receptor signaling is associated with a cellular stress response. ER stress induced by thapsigargin significantly downregulated c-MET in liver cells [53]. In hepatocellular carcinoma cells, ER stress resulted in a reduction of the protein and phosphorylation levels of p145 MET (c-MET tyrosine kinase B subunit) [43]. Furthermore, c-MET paracrine signaling mitigated ER-stress-induced damage in the renal cortex of animals exposed to a high-fat diet [54]. Lastly, c-MET must be N-glycosylated to function [44]. ER stress is known to reduce N-linked glycosylation of proteins [55, 56]. Increased c-MET signaling in *Chd8*^V986*/+^ mice (Fig. 6, cluster 3), which is predicted to occur if glycosylation is enhanced, further supports reduced ER stress and a reduced UPR response in aged *Chd8* mutant mice. Of possible relevance, mutations in c-MET are also associated with ASD risk in humans and with deficits in synaptic connectivity [57–60].

It is unclear if CHD8 regulates expression of ER stress genes directly, or if CHD8 indirectly affects the expression of ER stress genes, such as via neurodevelopmental downregulation of synaptic genes. Whether these late onset transcriptomic changes reflect cumulative damage caused by *Chd8* haploinsufficiency throughout life or reflect changes caused by *Chd8* haploinsufficiency in adulthood will require further study with conditional knockout mice.

## Limitations

Several limitations should be considered while interpreting the results of our study. It is unclear whether the transcriptional changes observed with age in *Chd8*^V986*/+^ mice reflect a direct effect of CHD8-regulated gene expression or if CHD8 indirectly affects the expression of UPR/ER stress genes in adult mice as a consequence of neurodevelopmental abnormalities. Conditional knockout of *Chd8* in adult mice and genetic rescue experiments will be important to validate genotype-transcriptional profiling correlations; although conditional knockout studies do not genetically model lifelong *CHD8* haploinsufficiency in humans. Additionally, most gene expression changes were of small magnitude, consistent with prior transcriptomic studies of *Chd8* mutant mice, necessitating that pathway and network analyses be utilized to understand how *Chd8* haploinsufficiency affects the brain. No protein level validation was performed outside of CHD8 itself, thus correlating significant gene expression changes with protein levels requires further investigation. Lastly, our studies were focused on male mice because most humans with *CHD8* mutations are male. Future studies could be performed to evaluate whether similar brain transcriptomic and behavioral abnormalities present in embryonic and aged female *Chd8* haploinsufficient mice.

## Conclusions

Our study highlights the importance of evaluating the brain and behavioral phenotypes in *Chd8* mutant mice across the lifespan. While our study is focused on *Chd8*, our work suggests it may be important to study other ASD model mice across the lifespan, to evaluate the extent to which early neurodevelopmental deficits cause cascading brain and behavioral abnormalities later in life.

## Supplementary information

**Additional file 1.** Supplementary Data File 1: Sample and read depth

**Additional file 2.** Supplementary Data File 2: Differentially expressed genes at each age

**Additional file 3:.** Supplementary Data File 3: The full lists of genes in each cluster

**Additional file 4.** Supplementary Data File 4: Gene ontology/pathway analyses

**Additional file 5.** Supplementary Data File 5: Very few differentially expressed genes shared across other Chd8 mouse models, even when comparing similar ages

**Additional file 6.** Supplemental Information

## Data Availability

The sequencing data generated during this study are available at GEO under accession GSE142208.

## Abbreviations

ASD: Autism spectrum disorder
ATF4: Activating transcription factor
ATF6: Activating transcription factor 6
*CHD8*: Chromodomain helicase DNA-binding protein 8
E14.5: Embryonic day 14.5
ER: Endoplasmic reticulum
GRP78: 78-kDA glucose-regulated protein
HGF: Hepatocyte growth factor
HSF1: Heat shock factor 1
IRE1: Inositol-requiring enzyme
mTORC1: Mammalian target of rapamycin complex 1
PERK: Protein kinase-like ER kinase
phospho-S6: Ribosomal protein S6
UPR: Unfolded protein response
WT: Wild-type
XBP1: X-box binding protein 1
