# Correction to: *Chd8* haploinsufficiency impairs early brain development and protein homeostasis later in life

**DOI:** 10.1186/s13229-021-00438-6

**Published:** 2021-05-08

**Authors:** Jessica A. Jiménez, Travis S. Ptacek, Alex H. Tuttle, Ralf S. Schmid, Sheryl S. Moy, Jeremy M. Simon, Mark J. Zylka

**Affiliations:** 1 Curriculum in Toxicology and Environmental Medicine, The University of North Carolina at Chapel Hill, Chapel Hill, NC 27599 USA; 2 UNC Neuroscience Center, The University of North Carolina at Chapel Hill, Chapel Hill, NC 27599 USA; 3 Carolina Institute for Developmental Disabilities, The University of North Carolina at Chapel Hill, Chapel Hill, NC 27599 USA; 4 Department of Psychiatry, The University of North Carolina at Chapel Hill, Chapel Hill, NC 27599 USA; 5 Department of Genetics, The University of North Carolina at Chapel Hill, Campus Box #7264, Chapel Hill, NC 27599 USA; 6 Department of Cell Biology and Physiology, The University of North Carolina at Chapel Hill, Chapel Hill, NC 27599 USA

## Correction to: Molecular Autism (2020) 11:74 10.1186/s13229-020-00369-8

Following publication of the original article [1], the authors identified some errors in Supplementary files as Supplementary data file 2 and Supplementary data file 5 were mixed up. This mixup has been corrected.
