# Supplementary material for: Chd8 haploinsufficiency impairs early brain development and protein homeostasis later in life
Source: Mol Autism. 2020 Oct 5;11:74. doi: 10.1186/s13229-020-00369-8 (PMC7537101; doi:10.1186/s13229-020-00369-8)
Supplement: Supplementary file 6 — Additional file 6. Supplemental Information [file 13229_2020_369_MOESM6_ESM.docx]

**Supplementary Information** (Jiménez et al.)

**Supplementary Figure 1.** Percentage of mouse stranger dyad samples showing tube co-occupancy (Co-occ.), single occupancy (Single), and no occupancy (vacant), sampled every 2 minutes over a 60 minute observation period. Data represents means ± S.E.M. of 12 WT and 10 *Chd8^V986*/+^* dyads. Equal numbers of male and female dyads were tested. *, p < 0.05.

**Supplementary Figure 2**. Boxplots of *Chd8* expression by age for WT and *Chd8^V986*/+^* mice. Points indicate expression values for individual samples. FDR adjusted p-value (WT versus *Chd8^V986*/+^*) is shown for each time point.

**Supplementary Figure 3**. Plots show the mean expression (across replicates) of all genes associated with the pathways shown in Figure 5. Shaded areas are +/- one standard deviation between replicates. Data from WT and *Chd8^V986*/+^* samples are plotted separately.

**Supplementary Figure 4**. Boxplots of *Xbp1* expression by age for WT and *Chd8^V986*/+^* mice. *. Points indicate expression values for individual samples. FDR adjusted p-value (WT versus *Chd8^V986*/+^*) is shown for each time point.

**Supplementary Figure 5.** Differentially expressed (A) down regulated and (B) up regulated genes that are unique (single black dot) to a single *Chd8* mouse model or shared (black dots connected by lines) between one or more *Chd8* mouse models. The number of genes is shown above each bar. The age at which samples were collected is indicated. The FDR adjusted p-value of the overlap of up and down regulated genes by time point between this study’s model, and the other models is shown in (C).

**Supplementary Data File 1.** Read depth (reads passing filtering parameters that aligned to the reference genome) for each sample.

**Supplementary Data File 2**. Normalized expression and differential expression testing (WT versus *Chd8^V986*/+^*) results for all genes in E14.5, 1, 6, and 12 month cortical samples.

**Supplementary Data File 3**. Genes in Clusters 1-5.

**Supplementary Data File 4**. Functional pathways that are significantly enriched in Clusters 1-5. Pathway sources: Kyoto Encyclopedia of Genes and Genomes (KEGG), Gene Ontology:Biological Process (GO:BP), Gene Ontology:Molecular Function (GO:MF), Gene Ontology:Cellular Component (GO:CC), Reactome (REAC).

**Supplementary Data File 5.** List of differentially expressed genes that are unique or shared between one or more *Chd8* mouse models. The numbers in this data file correspond to the numbers shown in Supplementary Figure 5 for up regulated (up) and down regulated (down) genes.

**Supplementary Figure 1.**


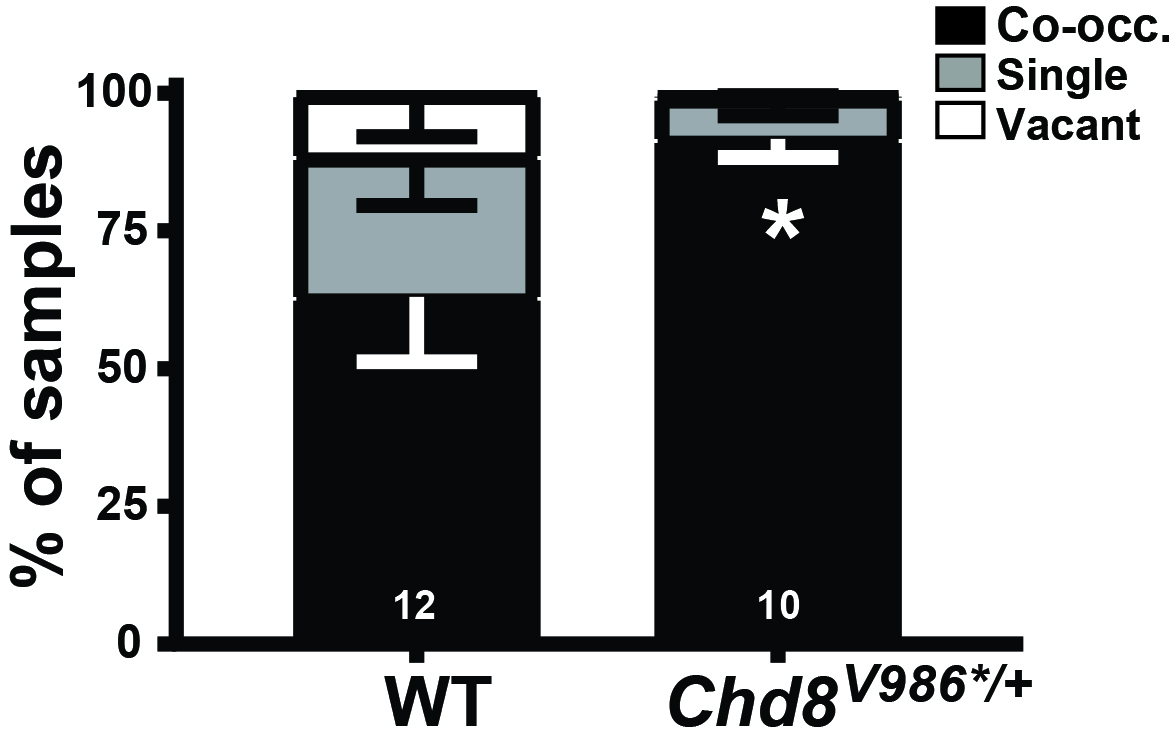


**Supplementary Figure 2.**

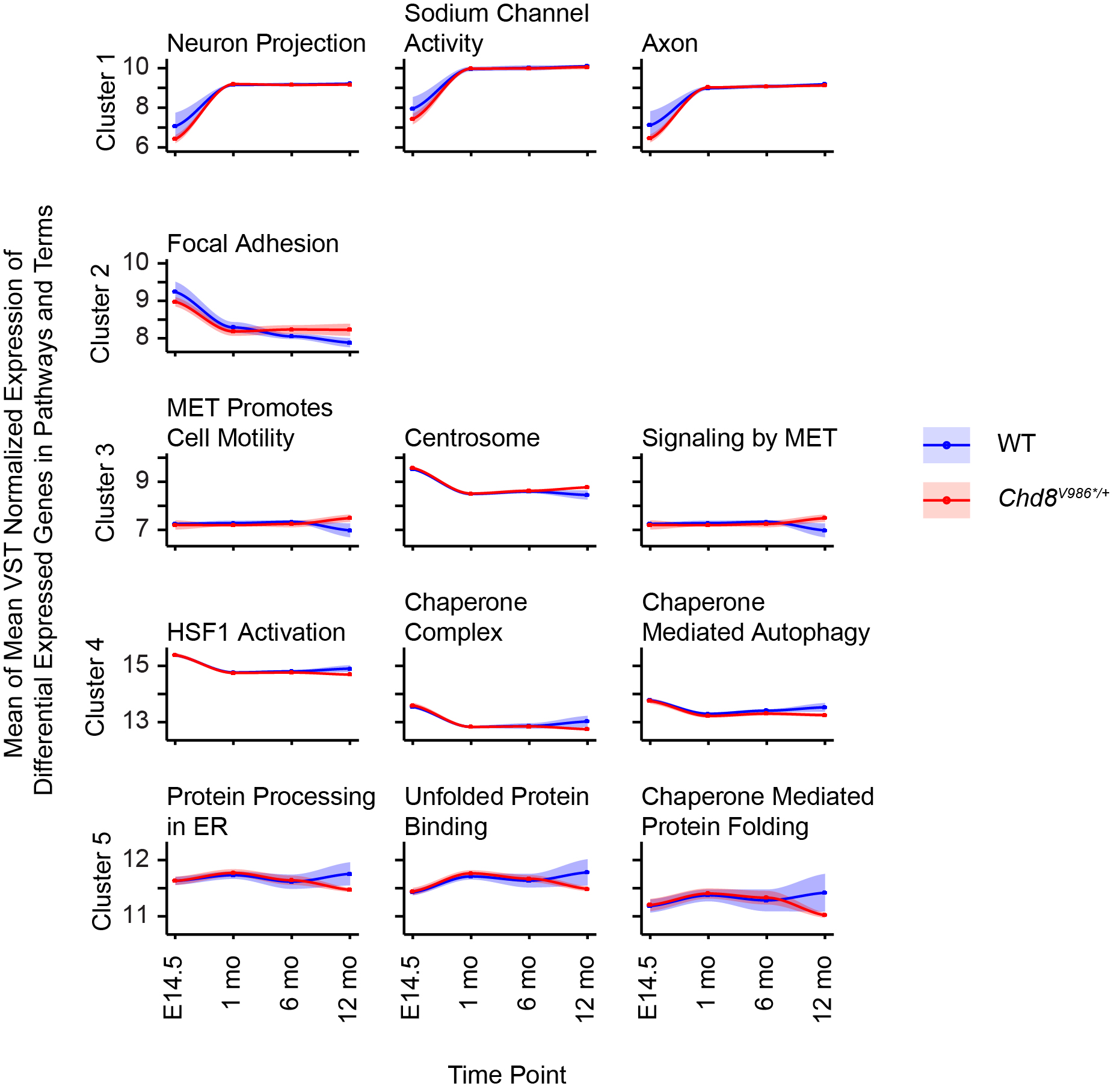
**Supplementary Figure 3.**

**Supplementary Figure 4.**

**Supplementary Figure 5.**

**
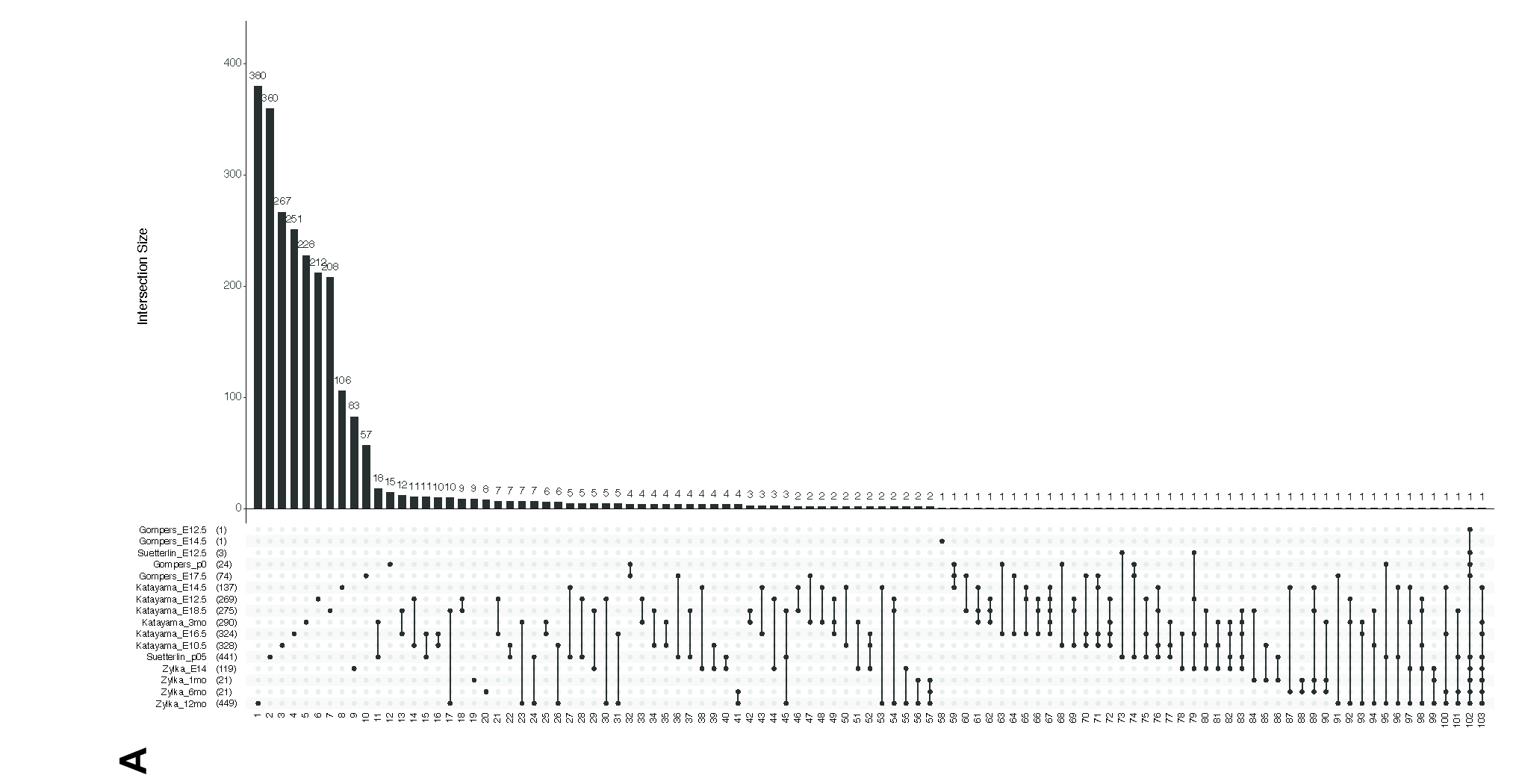
**

**
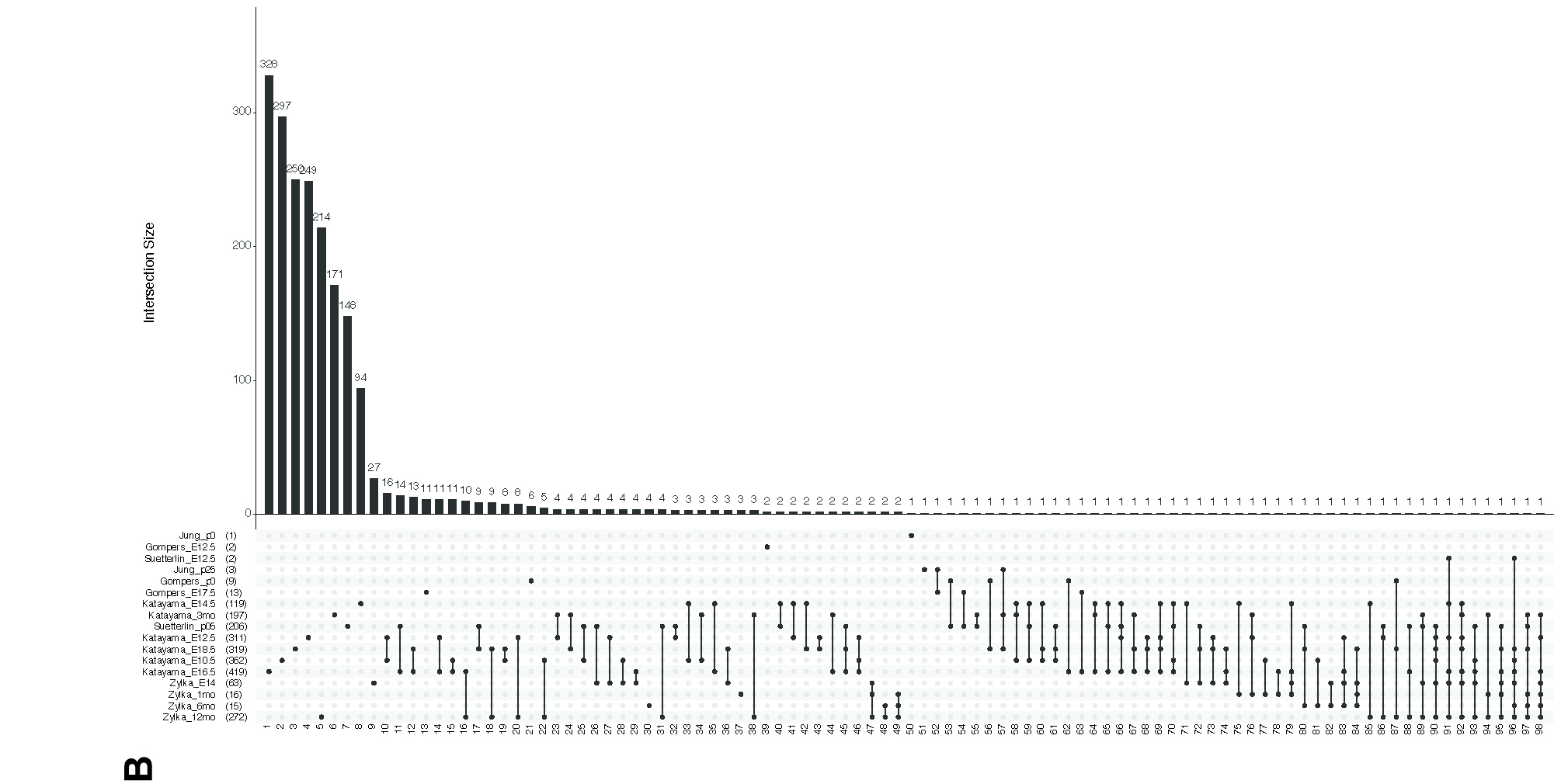
**

**
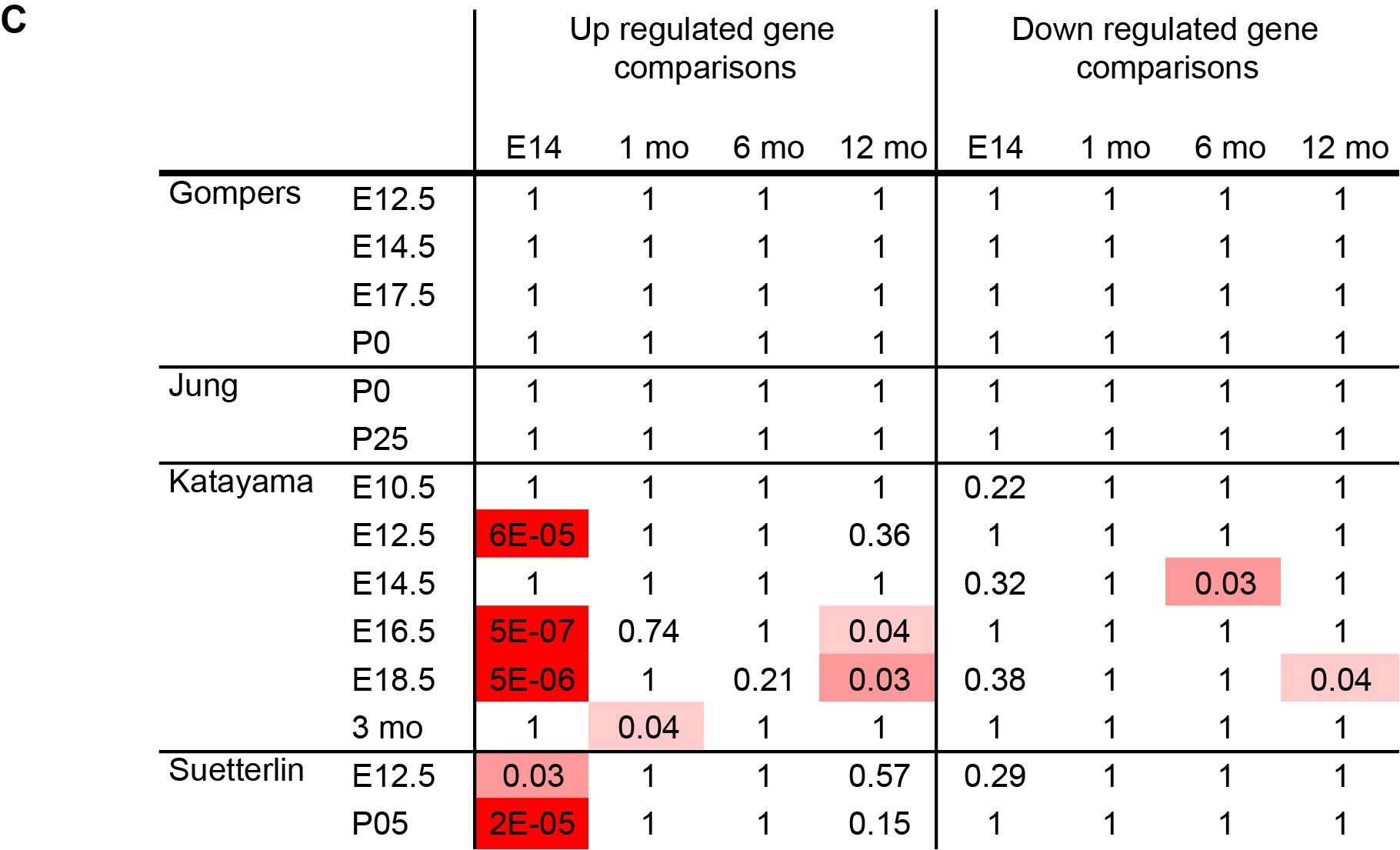
**
